# Bilateral breast cancer among three Yoruba women in a Nigerian teaching hospital

**DOI:** 10.1259/bjrcr.20150156

**Published:** 2015-08-11

**Authors:** Millicent Olubunmi Obajimi, Adenike Temitayo Adeniji-Sofoluwe, Adewunmi Oluseye Adeoye, Gbolahan Oladele Obajimi, Mustapha A Ajani, Prisca Olabisi Adejumo, Omolola M Akinwunmi

**Affiliations:** ^1^ Department of Radiology, College of Medicine, University of Ibadan, Ibadan, Nigeria; ^2^ Department of Radiology, University College Hospital, Ibadan, Nigeria; ^3^ Department of Pathology, College of Medicine, University of Ibadan, Ibadan, Nigeria; ^4^ Department of Pathology, University College Hospital, Ibadan, Nigeria; ^5^ Department of Obstetrics & Gynaecology, College of Medicine, University of Ibadan, Ibadan, Nigeria; ^6^ Department of Obstetrics & Gynaecology, University College Hospital, Ibadan, Nigeria; ^7^ Department of Nursing, College of Medicine, University of Ibadan, Ibadan, Nigeria; ^8^ Department of Nursing, University College Hospital, Ibadan, Nigeria

## Abstract

Breast cancer is the most common cancer among females in Nigeria. Bilateral breast cancer can occur synchronously or metachronously. We report three different cases of bilateral breast cancer in three female patients managed by the Ibadan Multidisciplinary Breast Tumour Board, domiciled at the University College Hospital (UCH), over a 3-year period. Two of these patients had synchronous bilateral breast cancer and developed cancer in the second breast during the course of management. These case reports may therefore stimulate further research on the clinicopathological features and the progression of bilateral breast cancer among females, especially in our environment. Our patients were premenopausal and the immunochemistry of the tumours showed a triple-negative immunophenotype. The other features of presentation, investigation, diagnosis and follow-up care are the highlights of this presentation.

## Summary

Breast cancer is the most common cancer among females in Nigeria. Bilateral breast cancer can occur synchronously or metachronously. We report three different cases of bilateral breast cancer in three female patients managed by the Ibadan Multidisciplinary Breast Tumour Board (MDT), domiciled at the University College Hospital (UCH), over a 3-year period. Two of these patients had synchronous bilateral breast cancer and developed cancer in the second breast during the course of management. These case reports may therefore stimulate further research on the clinicopathological features and the progression of bilateral breast cancer among females, especially in our environment. Our patients were premenopausal and the immunochemistry of the tumours showed a triple-negative immunophenotype. The other features of presentation, investigation, diagnosis and follow-up care are the highlights of this presentation.

Breast cancer is the most common female malignancy all over the world including Nigeria.^1–4^ Among Caucasians, it is more common in the middle and older age groups than in young females.^3^ But reports in Nigeria show that breast cancer is more common in the premenopausal age group.^4–6^ Evidence abound that bilateral breast disease is common in the Caucasian community.^7,8^ The actual prevalence of bilateral breast cancer in our setting is unknown; this is owing to poor data and under-reporting.

Bilateral breast cancer has an overall incidence of 4-–20% in patients with operable breast cancer.^9^ The risk factors associated with bilateral occurrence include: a positive family history of breast cancer in a first-degree relative, young age at diagnosis of primary breast cancer, histologically diagnosed invasive lobular carcinoma of the initial breast mass lesion, multicentricity and previous history of exposure to ionizing radiation.^2^

This is a report of three premenopausal females with bilateral breast cancer managed by the MDT between 2009 and 2012. Two out of the three patients had primary unilateral breast mass at presentation but developed bilateral (synchronous) disease during the course of the management, while the third had bilateral disease at her first presentation.

The epidemiology, clinical presentation, diagnosis and management of bilateral breast cancer in the three Nigerian female patients is hereby reported.

## Case report one

OD is 28-year-old premenopausal, para 1 +0 Yoruba female who presented at the age of 28 years for sonographic examination of a recurrent left breast mass. She underwent a lumpectomy 3 months earlier at another tertiary facility with a histological diagnosis of invasive ductal carcinoma (IDC) of the excised mass. She has a strongly positive family history of breast cancer in first-degree relatives (her mother and maternal grandmother). The details of presentation and the death of her grandmother were not disclosed. However, her mother was diagnosed at the age of 52 years and died 6 years later of the disease. The patient presented with bloody left nipple discharge. A clinical breast examination was performed before sonomammography. This revealed a scar at the upper outer quadrant of the left breast, consistent with the site of the previous lumpectomy. There was a palpable, firm retroareolar mass in the same breast that was fairly mobile with associated thickening of the areola. There was also bloody nipple discharge and ipsilateral axillary lymphadenopathy. At the time of the examination, the right breast was essentially within normal limits.

Left sonomammography performed at the Radiology Department, UCH, with the Logiq P5 GE ultrasound machine (GE Healthcare, Waukesha, WI) using the high frequency linear transducer (10 MHz) showed a mixed density mass with specks of calcifications at the 3 o’clock position and in the retroareolar region. The overlying areola was thickened and there was architectural distortion from the previous scar. Also, there were two axillary lymph nodes with fatty replaced hila. A final BI-RADS assessment of category 5 (highly suggestive of malignancy) was made, with possible invasion of the ipsilateral axillary nodes. An immediate ultrasound-guided core biopsy of the mass was performed and histological examination confirmed IDC, Scarff–Bloom–Richardson grade 2, score 6; the immunochemistry result was triple-negative.

She was immediately commenced on four courses of adriamycin and cyclophosphamide neoadjuvant chemotherapy and later had left modified radical mastectomy. She also had four courses of radiotherapy and paclitaxel adjuvant chemotherapy a few months after the left mastectomy.

**Figure 1.**
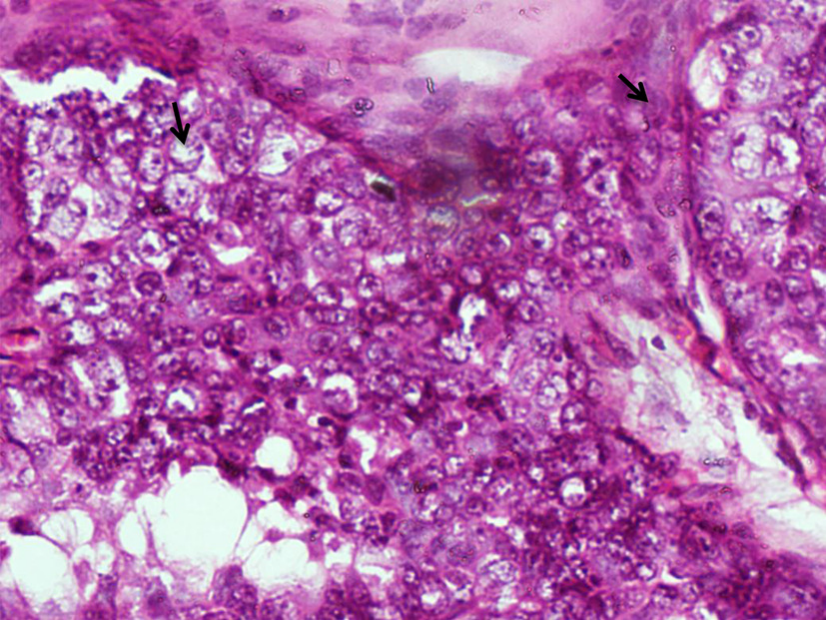
Histopathological specimen from left mastectomy showing moderately pleomorphic cells with large vesicular nuclei and prominent nucleoli (arrows) consistent with invasive ductal carcinoma (haematoxylin and eosin × 400).

She made progress and resumed work. She also got married 2 years later and became pregnant immediately after. During cyesis, she developed another lump on the contralateral side. A breast ultrasound was performed and showed evidence of architectural distortion at the 6 o’clock position but no definite intramammary mass was seen. There were, however, enlarged ipsilateral replaced axillary nodes. An impression of a contralateral tumour was made and a final BI-RADS category of 4c was assigned to the study. An immediate ultrasound-guided core needle biopsy (CNB) of the suspicious area revealed malignant features (Figure 1). She, however, declined chemotherapy until after the delivery of her baby; she was admitted for close monitoring, further investigations and palliative care. At term, she was delivered of a normal male infant by spontaneous vaginal delivery. Unfortunately, she could not breastfeed the baby as she re-presented 4 weeks after delivery at the Accident and Emergency Unit, UCH, owing to weight loss, breathlessness and progressive non-productive cough of 2 weeks’ duration. Further investigations at the time showed widespread canon-ball opacities consistent with metastases in both lung fields with bilateral pleural effusion consistent with pulmonary metastasis. A bone scan also confirmed widespread bone metastasis. Abdominopelvic ultrasound found metastasis to the liver. An impression of a rapidly progressing disease was made.

During the most recent admission, she was initially managed conservatively, counseled on family planning and offered six courses of adjuvant chemotherapy. It is important to note that, throughout her management, she enjoyed the strong social support of her family members and her husband.

## Case report two

KA is a 50-year-old premenopausal parous female who presented for imaging at the age of 45 years with a history of a right breast lump of 6 months’ duration. She has no positive family history of breast cancer.

**Figure 2.**
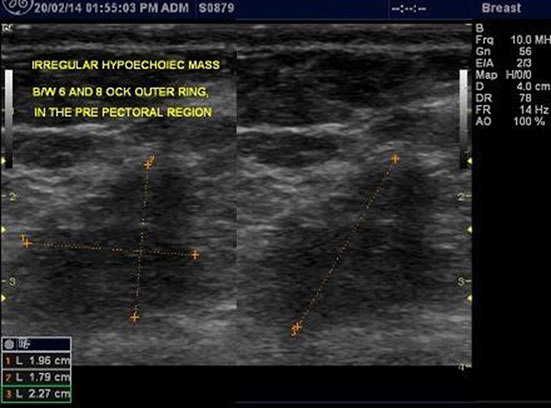
An orthogonal sonomammographic scan of the right breast at first presentation showing a poorly circumscribed, taller than wide, hypoechoic mass located at the 12 o’clock position.

**Figure 3.**
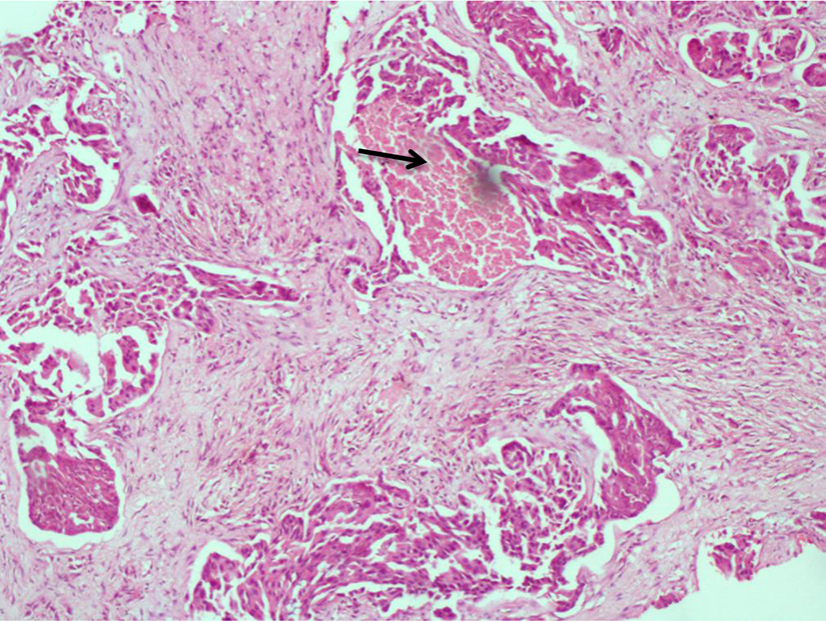
Histopathological specimen of ultrasound-guided core biopsy of the right breast mass showing invasive nests and cords of moderately pleomorphic epithelial cells with hyperchromatic nuclei and eosinophilic cytoplasm with an area of comedonecrosis (arrow) consistent with invasive ductal carcinoma (haematoxylin and eosin × 100).

Right sonomammography performed at the Radiology Department, UCH, with a Logiq P5 GE ultrasound machine using the high frequency linear transducer (10 MHz) at her first presentation showed a poorly circumscribed mixed echogenic mass at the 12 o’clock position measuring 4.9 × 3.2 cm. The mass showed specks of calcifications within it. The Doppler interrogation showed evidence of increased vascularity. A final BI-RADS category of 4 (suspicious for malignancy) was assigned to the study (Figure 2). Conventional mammography performed in the same department confirmed a poorly defined mass with microcalcifications and architectural distortion at the 12 o’clock position. The left breast at the time of the study was normal on both imaging modalities. She subsequently had an ultrasound-guided CNB of the right breast lump and the histopathological report confirmed IDC (Figure 3). The immunochemistry report was triple-negative. She immediately commenced neoadjuvant chemotherapy and later had modified right radical mastectomy. After surgery, she had adjuvant chemo- and radiotherapy. She was a compliant patient who had remission for approximately 5 years; during this period, she was off chemotherapeutic drugs.

She developed a painless progressive lump in the contralateral breast 5 years later and presented to the surgical outpatient clinic. On examination, there was a palpable left breast mass with associated bloody nipple discharge, which was confirmed on sonomammography. Histological examination of the ultrasound-guided CNB specimen of the contralateral mass confirmed it to be an IDC. She then had modified left radical mastectomy after adjuvant chemotherapy and has since been placed on paclitaxel and radiotherapy.

## Case report three

OA is a 36-year-old premenopausal para 2 + 0 Yoruba female who presented in 2013 at the General Outpatient Department, UCH, owing to a palpable painless right breast lump of 3 years’ duration. She had a positive family history of breast cancer in a first-degree relative (her mother) who was diagnosed at the age of 45 years and died at the age of 55 years. At presentation, she was found to be lactating and there was a firm, non-mobile nodular mass in the lower outer quadrant (LOQ) of her right breast. A similar but smaller mass was palpated in the left breast. There were no associated skin changes or nipple retraction.

**Figure 4.**
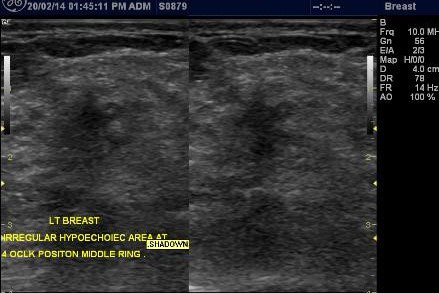
Ultrasound of the breast in orthogonal views showing a spiculated, poorly circumscribed, hypoechoic mass with posterior acoustic shadow in the left breast.

**Figure 5.**
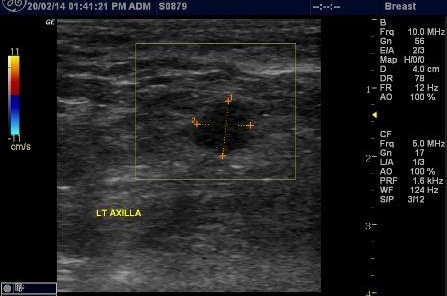
Ultrasound of the axilla with a replaced node showing an enlarged lymph node with irregular margins and loss of the usual corticomedullary differentiation; however, Doppler interrogation showed no increased vascularity.

Because she was lactating, a conventional mammography could not be performed; however, a sonomammography was requested and this was performed at the Radiology Department, UCH, with a Logiq P5 GE ultrasound machine using the high frequency linear transducer (10 MHz) and it confirmed bilateral disease. The lesions found were two poorly circumscribed hypoechoic masses with spiculated margins in the LOQ at the 6- –9 o’clock position of the right breast; one deep in the prepectoral region and the other superficial in the middle ring of the right breast between the 6 and 8 o’clock position. They measured 4.3 × 2.9 cm and 1.4 × 1.3 cm, respectively. The latter mass showed specks of calcifications within it. Both masses in the right breast showed posterior acoustic shadows. There was also associated architectural distortion. The contralateral (left) breast also showed a similar but smaller mass at the 4 o’clock position in the middle ring of the breast. There were multiple enlarged lymph nodes with replaced hila in both axillae (Figures 4 and 5). One of the nodes in the right axilla showed foci of calcifications and measured 1.4 × 1.5 cm. An impression of bilateral breast masses was made and a final BI-RADS category of 5 (highly suggestive of malignancy) was assigned to the study.

She subsequently had ultrasound-guided CNBs performed on both breast masses and a histological diagnosis of bilateral IDC was made. The immunochemistry report was triple-negative. Fine needle aspiration was also performed on a palpable right supraclavicular lymph node and the histological report confirmed benign features.

Neoadjuvant chemotherapy was then offered while being worked up for bilateral mastectomy; she rejected both chemotherapy and surgery despite all the counsel given on the need and benefits of early intervention. She opted for alternative therapy owing to the lack of funds.

## Discussion

Breast cancer is known to be the leading female malignancy in the world and the most common malignancy affecting Nigerian females.^4,10,11^ Although the incidence of breast cancer appears to be stabilizing or decreasing in some Western countries, the breast cancer burden has steadily increased in many developing countries such as Nigeria, which previously had low incidence rates; the relatively active lifestyle, attributed to late menarche, prolonged the period of breastfeeding and diet.^12^ However, the current trend of westernization of dietary habits and lifestyle is changing the demographic and socioeconomic profile of the country at large.^10,13^ Nevertheless, the incidence of breast cancer in Western countries is about sixfold higher than the developing countries of Africa and Asia owing to demographic factors such as longer life expectancy, better and early reporting of cases and easy access to healthcare.^4^

Epidemiologic studies have identified a number of risk factors that are associated with an increased risk of developing breast cancer. These include increasing age, a positive history of breast cancer in first-degree relatives, late age at first full-term pregnancy, nulliparity, early age at menarche, late age of menopause, alcohol, smoking, obesity and sedentary lifestyle.^14^ Moreover, young age at diagnosis (usually younger than 40 years), positive family history of breast cancer and previous exposure to ionizing radiation have been reported to be established risk factors in the development of bilateral breast cancer.^7^ The mean age of bilateral breast malignancy in a study by Aghadiuno et al ^15^in UCH on 18 females was 30 years. All our patients were premenopausal with age ranging between 32 and 50 years. All of them belonged to the middle class and had tertiary education.

Bilateral breast malignancy can be synchronous or metachronous. Synchronous breast cancer has been defined as cancer diagnosed in both breasts simultaneously or within 3 months of diagnosis of the first tumour. Metachronous breast cancer has been defined as diagnosis of cancer in the contralateral breast more than 3 months after the diagnosis of the tumour in the first breast.^7^ Two of our patients had metachronous breast cancer while the third had synchronous breast cancer.

A positive family history of breast cancer in a first-degree relative is the most widely recognized breast cancer risk factor, as observed in two of the cases discussed. In addition, females with a family history of cancer syndromes such as ovarian, tubal and peritoneal cancers have increased risks for mutation in the breast cancer susceptibility genes *BRCA*1 or *BRCA*2. However, genomic studies are yet to be well established in our setting.

Clinically, breast carcinomas are mostly asymptomatic in the early part of the course of the disease. But larger tumours may present as painless palpable breast masses, as seen in the patients discussed. Our patients also presented with skin changes, nipple/skin retraction, bloody nipple discharge (except for the lactating patient) and axillary lumps, which are all pointers to late presentation; a feature of breast cancer in the developing world. Poor awareness on breast cancer care and the lack of national screening guidelines and programmes, and poor infrastructure, all contribute to this well-documented late presentation and difficult breast cancer management. Breast cancer commonly metastasizes through the lymphatic drainage to the axillary and internal mammary lymph nodes, liver, lungs, bone and brain. Two of our patients showed tumour-replaced nodes at presentation, another evidence of late presentation in the cases reported. These worsen the prognosis of the patient. One of our patients had distant metastasis to the liver, lungs and long bones.

Breast cancer is also diagnosed during pregnancy and in the postpartum period and tends to affect females in their mid-30s. The hormonal changes during pregnancy is known to accelerate the growth of breast cancer.^16^ This must have accounted for the rapid progression of our first case who was diagnosed with cancer in the left breast, then got pregnant during the course of treatment and later developed contralateral breast cancer and distant metastasis, which were all diagnosed during her gestation, consistent with rapid progression of the disease with pregnancy. Lactation, however, has previously been shown to be protective against breast cancer.^14,17^ This is at abeyance with our third patient who presented during lactation and had a positive family history.

Mammography is still the gold standard for screening breast diseases in females, especially those aged over 40 years in whom fatty evolution of the breasts makes them mammographically transparent. However, in the younger females with mammographically dense breasts, ultrasound and MRI are recommended. All our three patients had breast ultrasound, which, apart from characterizing the tumour, also showed malignant features that correlated with the mammographic findings. It also prevented irradiation of the virgin breast in the 28-year-old female. Breast ultrasound is invaluable in guided biopsies in such females who in a setting like ours have no access to stereotactic or MR-guided biopsy.

The knowledge of the importance of breast cancer screening and the early detection measures of breast cancer have been reported to be poor in both the rural and urban settlements in Nigeria.^18^ A study performed by Obajimi et al^19^ further reiterated this poor awareness among females attending the General Outpatient Clinic, UCH. The above may partly be owing to the level of education and socioeconomic status of females, the poor knowledge of mammography even among the health workers and the low resource setting of the country at large.^19^

All our patients had histologically diagnosed IDC, which is the most common type of histologically diagnosed breast cancer. In Nigeria, the incidence of other histological types that include lobular carcinoma *in* *situ,* IDC, infiltrating lobular carcinoma, medullary carcinoma, mucinous carcinoma, tubular carcinoma, papillary carcinoma, metaplastic breast cancer and mammary Paget disease are not as common.^5^

Research in the USA showed that estrogen receptor (ER)- and progesterone receptor-positive breast cancers represented the majority of breast tumours, although proportions varied with ethnicity or race. It was most common among the non-Hispanic whites followed by Hispanics then followed by the African American population.^20^ Recent studies performed on Nigerian females showed that only 25% of breast cancers were ER positive.^21^ According to a study by Bauer et al,^22^ triple-negative breast cancers found in our three patients affect more females in the low socioeconomic group. They also had worse prognosis, which would have accounted for the rapid progression of the disease in the first female in particular who was only 28 years old at presentation and developed distant metastasis within 2 years in spite of immediate treatment.

Globally, the treatment of breast cancer is now personalized, depending on the histological type, stage of disease, immunochemistry drug preference and progress. In settings with late presentation such as ours, neoadjuvant therapy is administered before mastectomy and adjuvant chemotherapy, unlike developed countries where the goal is conservative surgery, especially in young patients.

The risk factors associated with bilateral disease include a positive family history. Two of our patients had a positive family history in the first-degree relative; the younger of the two also had a history in her maternal grandmother. Other factors are early age at diagnosis, common to all our three patients, and multicentricity of the tumour. Although bilateral breast cancer may be explained by non-genetic factors, the genetic factors that have been implicated in breast cancer include *BRCA*1 and *BRCA*2 mutations. Lee et al^23^ reported a positive family history of breast cancer in 28% of females with unilateral breast cancer and up to 40% in patients with bilateral breast cancer. It has also been observed that up to 70% of females diagnosed with other malignant tumours have a first-degree relative affected by breast cancer.^23^

Good patient outcome and improved quality of life is the goal of the MDT. Both our patients who received therapy are success stories. The huge social support provided by relatives, encouraged by the stakeholders of the MDT, as well as the personal touch in their follow-up contributed immensely to the progress reported in these patients to date.

## Conclusions

These case series suggest a possible increase in the incidence of bilateral breast cancer in females in Ibadan. As illustrated in Table 1, some of the cases occurred during the management of breast cancer. The case reports of these three patients will serve as a template for a future review of a larger cohort in the same setting.

**Table 1.** Summary of the cases: age at clinical presentation, tumour grade, histology, ER/PR negative, treatment and recommendations.

	CASE 1	CASE 2	CASE 3
Age (years)	28	50	36
Presentation	Recurrent left breast mass of 3 months' duration. Developed right breast mass 2 years later during cyesis	Patient with known breast cancer of the left breast developed right breast mass of 6 months' duration	Lactating mother with bilateral breast masses of 3 years' duration
Tumour grade	SBR grade 2, score 6	SBR grade 2, score 6	SBR grade 2, score 6
Histology	IDC	IDC	IDC
ER/PR status	Negative	Negative	Negative
Treatment	AC neoadjuvant chemotherapy, left breast radiotherapy, paclitaxel adjuvant chemotherapy postoperatively	Neoadjuvant chemotherapy with modified radical right mastectomy	EC chemotherapy. She was booked for bilateral mastectomy before she opted for alternative therapy
Recommendation	To continue palliative care	Continue management and follow-up at surgical outpatient clinic	For intensive counseling sessions

AC, adriamycin and cyclophosphamide; EC, epirubicin and cyclophosphamide; ER, estrogen receptor; IDC, invasive ductal carcinoma; PR, progesterone receptor; SBR, Scarff–Bloom–Richardson.

## Learning points

1. Patients with a positive family history of breast cancer should have early screening for breast cancer.
2. Any female with unilateral breast cancer must be examined and fully evaluated for contralateral breast cancer and metastatic disease; the importance of a multidisciplinary approach can also not be overemphasized.
3. Pregnant females with breast cancer should be well informed about the importance of close monitoring of both their unborn babies and the co-morbidity, breast cancer.
4. The family members of every patient being managed for breast cancer must be well informed and carried along with the patient's management to improve family and social support, which are crucial for healing the patient; also the managing team should help in alleviating the fears/anxieties in the patients and their loved ones.
